# Proceedings of the Central New York Homœopathic Medical Society

**Published:** 1887-10

**Authors:** T. Dwight Stow

**Affiliations:** Secretary pro tem.


### PROCEEDINGS OF THE CENTRAL NEW YORK HOMŒOPATHIC MEDICAL SOCIETY.

The annual meeting of the Central New York Homœopathic Medical Society was held in the office of Dr. Wm. A. Hawley, in the city of Syracuse, Thursday, June 16th, 1887. The meeting was organized by making Dr. A. J. Brewster Chairman *pro tem.*, and Dr. T. D. Stow, Secretary *pro tem.*, the President, Dr. Biegler, and the Secretary, Dr. Schmitt, being absent. Dr. Hawley showed a letter from Dr. Smith, stating that Dr. Biegler had an attack of hemiplegia, which would prevent his attendance.

In the absence of the records, the minutes of last meeting were not read.

Members present: Drs. Wm. A. Hawley, A. J. Brewster, Stephen Seward, R. S. True, H. Dada Emens, G. H. Greeley, of Syracuse; T. L. Brown, of Binghamton; Leslie Martin, of Baldwinsville; T. D. Stow, of Mexico.

Dr. Hawley presented the application of Dr. R. S. True, of Syracuse, for membership.

No communication from the President. No report of censors. No election of new members. Reading of *Organon* was dispensed with.

Unanimous sympathy was expressed for the President, Dr. J. A. Biegler, who is absent by reason of a sudden but partial stroke of paralysis. Regrets were also expressed for the sickness of Dr. L. B. Wells, of Utica.

Reports on medical subjects being in order, Dr. Stow read a paper on the ætiology and pathology of syphilis, the subject chosen for discussion at the March meeting. In addition to his paper, he cited facts taken from the publications of the "London Society for the Abolition of Compulsory Vaccination," kindly furnished by William Young, Secretary of that Society, showing that syphilis is inoculable not only by impure coition but by vaccination, whether of human or bovine lymph. He argued that if syphilis is to be avoided and dreaded, then its transmission through vaccination—which is a well-established fact—is equally reprehensible, and physicians in general, *but homœopathic physicians in particular*, ought to discountenance the practice. Dr. Stow also distributed documents relating to the question, kindly furnished by Mr. William Young, 77 Atlantic Road, Brixton, England.

The meeting then adjourned till two P. M. Reassembled at two P. M., and called to order by Chairman Brewster.

Dr. Leslie Martin, of Baldwinsville, then read a paper on syphilitic poisoning by inoculation of his finger while attending a case of labor. He gave a pathological description of his case that was very interesting, and stated how and by what remedy he was cured. The principal remedies were Calc. carb., Silicea, Nit. acid, Staphisagria, and Thuja, according to their indications.

Considerable wordy sparring took place during the reading of the two papers.

Dr. Hawley, in the absence of any paper furnished by Dr. Kent, of St. Louis, opened the discussion by reporting the treatment of a case of syphilitic iritis, a very interesting report showing the metastatic nature of syphilitic germs, the beauty of action of the homœopathic remedy in a minimum quantity. His remarks called out questions and desultory statements, not in the recollection of the scribe to record.

Dr. Brown, of Binghamton, followed Dr. Hawley, commenting on Dr. Hawley's case, sharply criticising the opposition of Dr. Stow to vaccination, and making some very fine suggestions as to the diet, cleanliness, and hygiene of syphilitic patients.

Dr. True cited a case requiring Aurum.

Dr. Seward stated his belief in vaccination, and the successful uses of original cow-pox virus he had taken from a cow.

Dr. Stow thought Dr. Seward's experience with cow-pox lymph was decidedly negative:

First. Because Dr. Jenner and his disciples long ago abandoned that practice as unreliable and worthless.

Second. Because only *milch* cattle of the bovine species have that simple malady, and because the suspicion is that when it does occur it is conveyed to the bag or teats of the cow by milkers, who thus convey nobody knows what!

Third. Because there is no evidence that virus, taken from the calf or cow, is freer from constitutional or other disease germs than humanized virus, and, further, that official statistics show that vaccination neither mitigates nor prevents small-pox.

Miscellaneous business being next in order, Dr. Brown, of Binghamton, made a speech laudatory of hygiene. He declared that one-half or two-thirds of the maladies we are called upon to treat are self-induced, the result of ignorance of natural law or the willful violation of such law; that the abandonment of a pernicious habit should be the first move—Homœopathy stepping in to hasten and to establish recovery.

Dr. True followed Dr. Brown, citing cases of drugging and the causes that lead him to abandon allopathy.

Dr. Hawley told an interesting story apropos to the occasion, illustrating the old saw, that "all's well that *ends well.*"

A desultory conversation ensued, during which the meeting adjourned, the time and place of the next to be Thursday, September 15th, at Dr. Hawley's office in the city of Syracuse.

Subject for discussion: What cures?

On the whole, the meeting was an interesting and profitable one, only lacking in point of numbers, but this was accounted for on the basis of the sickness of such as our worthy President, Dr. Wells, of Utica, etc., and the further fact of the proximity of the annual session of the International Hahnemannian Association at Long Branch, N. J., making it impossible for many to attend this meeting; lastly, but few can conveniently leave their business at such time.

T. Dwight Stow, *Secretary pro tem.*

